# Multisensory Causal Inference in the Brain

**DOI:** 10.1371/journal.pbio.1002075

**Published:** 2015-02-24

**Authors:** Christoph Kayser, Ladan Shams

**Affiliations:** 1 Institute of Neuroscience and Psychology, University of Glasgow, Glasgow, United Kingdom; 2 Departments of Psychology and BioEngineering and the Interdepartmental Neuroscience Program, University of California, Los Angeles, Los Angeles, California, United States of America

## Abstract

At any given moment, our brain processes multiple inputs from its different sensory modalities (vision, hearing, touch, etc.). In deciphering this array of sensory information, the brain has to solve two problems: (1) which of the inputs originate from the same object and should be integrated and (2) for the sensations originating from the same object, how best to integrate them. Recent behavioural studies suggest that the human brain solves these problems using optimal probabilistic inference, known as Bayesian causal inference. However, how and where the underlying computations are carried out in the brain have remained unknown. By combining neuroimaging-based decoding techniques and computational modelling of behavioural data, a new study now sheds light on how multisensory causal inference maps onto specific brain areas. The results suggest that the complexity of neural computations increases along the visual hierarchy and link specific components of the causal inference process with specific visual and parietal regions.

## Abstract 2

How does our brain organize and merge multisensory information? A new study localizes the brain regions underlying sensory fusion and causal inference by combining neuroimaging and computational modelling.

## Introduction

Our brain is continuously faced with a plethora of sensory inputs impinging on our senses. At any moment we see, hear, touch, and smell, and only the coordinated interplay of our senses allows us to properly interact with the environment [1]. How the brain organizes all these sensory inputs into a coherent percept remains unclear. As shown in a new study by Rohe and Noppeney, important insights can be obtained by combining computational models with carefully crafted analysis of brain activity [2].

The brain needs to solve several computational problems to make sense of its environment. Besides the analysis of specific sensory attributes (for example, to segment a scene into its constituent objects), two critical problems involve the inputs to different senses. One is the “multisensory integration problem”: how information is synthesized (or fused) across the senses. For instance, speech perception usually relies on the integration of auditory and visual information (listening to somebody’s voice while seeing his or her lips move). This integration problem can be challenging, as each sense only provides a noisy and possibly biased estimate of the respective attribute [3,4].

In addition, the brain needs to solve the “causal inference problem” [5–7]: it has to decide which sensory inputs likely originate from the same object and hence provide complementary evidence about this and which inputs originate from distinct objects and hence should be processed separately. One example is at a cocktail party, where many faces and voices can make it a challenge to know who called our name. Another example is at a ventriloquist’s performance, where we attribute the voice to the puppet rather than the actor. In practice, the tasks of inferring the causal structure and of obtaining precise estimates of sensory attributes are highly intertwined, as causal inference depends on the similarity of different sensory features, while the estimate of each attribute depends on the inferred causal origin. For example, the association of a face and a voice depends on both the perceived location of each as well as the match between sematic, social, or physical attributes derived from faces and voices. Hence, solving the causal inference problem has to rely on a number of factors such as spatial, temporal, and structural congruency, prior knowledge, and expectations.

In the 19th century, von Helmholtz already noted that perception requires solving multiple inference problems [8]. Yet, laboratory studies on multisensory integration often avoid the causal inference problem by crafting multisensory stimuli that leave little doubt as to whether they provide evidence about the same sensory object. However, the brain mechanisms underlying multisensory perception in everyday tasks can probably only be understood by considering both the integration and causal inference problems [9]. Fortunately, the field of Bayesian perception has provided a conceptual framework in which both can be cast in unified statistical terms [10].

## Bayesian Approaches to Multisensory Perception

**Fig 1:**
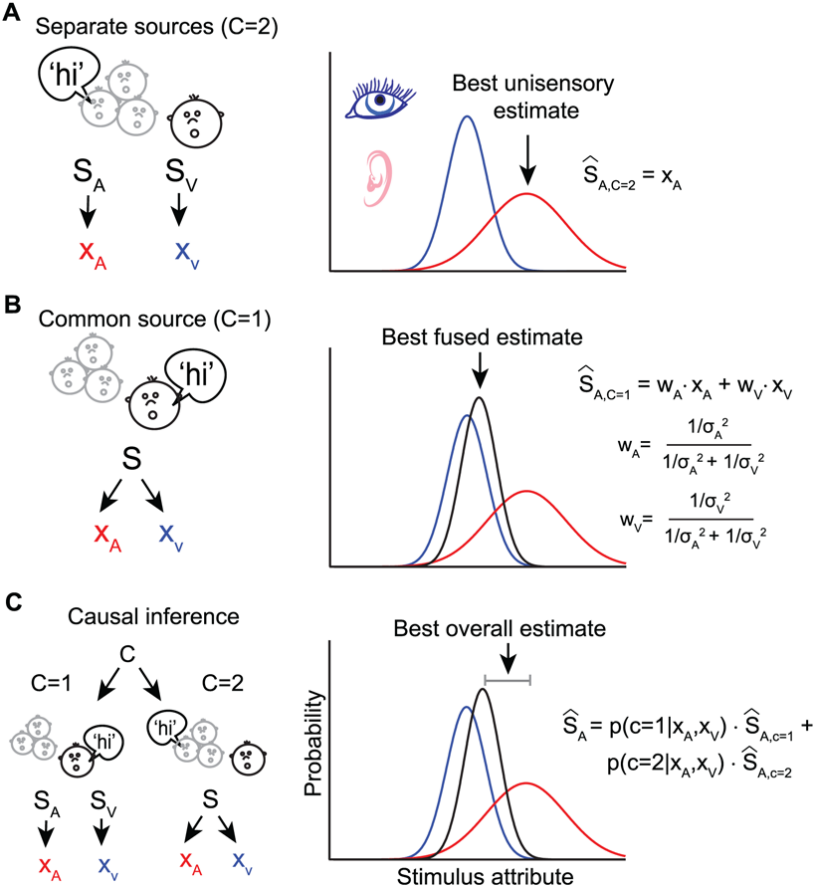
Bayesian models of multisensory integration. Schematic of different causal structures in the environment giving rise to visual and acoustic inputs (e.g., seeing a face and hearing a voice) that may or may not originate from the same speaker. The left panels display the inferred statistical causal structure, with S_A_, S_V_, and S denoting sources for acoustic, visual, or multisensory stimuli and X_A_ and X_V_ indicating the respective sensory representations (e.g., location). The right panels display the probability distributions of these sensory representations and the optimal estimate of stimulus attribute (e.g., location) derived from the Bayesian model under different assumptions about the environment. For the sake of simplicity of illustration, it is assumed that the prior probability of the stimulus attribute is uniform (and therefore not shown in the equations and figures). (A) Assuming separate sources (C = 2) leads to independent acoustic and visual estimates of stimulus location, with the optimal value matching the most likely unisensory location. (B) Assuming a common source (C = 1) leads to integration (fusion). The optimal Bayesian estimate is the combination of visual and acoustic estimates, each weighted by its relative reliability (with σ_A_ and σ_V_ denoting the inverse reliability of each sense). (C) In Bayesian causal inference (assuming a model-averaging decision strategy), the two different hypotheses about the causal structure (e.g., one or two sources) are combined, each weighted by its inferred probability given the visual and acoustic sensations. The optimal stimulus estimate is a mixture of the unisensory and fused estimates.

Bayesian statistics describes sensory representations in probabilistic terms, attributing likelihoods to each possible encoding of a sensory attribute [11]. Moreover, it describes how different variables interact in determining the outcome, such as how prior knowledge affects perceptual estimates or how inputs from two senses combine. As shown in Fig. 1A, when considered independently, each sensory modality can be conceptualized as providing a noisy (probabilistic) estimate of the same attribute. Yet, under the assumption of a common source, Bayesian inference predicts the multisensory estimate arising from the combination of both senses by weighing each input in proportion to its reliability (Fig. 1B).

This approach has provided invaluable insights about various aspects of perceptual multisensory integration [3,4,12,13] and helped to identify the sensory computations likely to be carried out by the underlying neural processes [14,15]. For example, studies on visual-vestibular integration have shown that the mathematical rules by which neural populations combine visual-vestibular information follow Bayesian predictions and that the relative weights attributed to each modality in the neural code scale with sensory reliability analogous to the perceptual weights [16].

## Probabilistic Models for Causal Inference

The Bayesian approach can be extended to model the causal inference problem by including inference about the environment’s causal structure (Fig. 1C). Depending on the task that the nervous system has to solve, different perceptual decision-making strategies can be used to derive estimates of sensory attributes based on the probabilities of each possible causal structure [17]. For example, when trying to minimize the error in the perceptual estimate, e.g., to precisely localize a speaker, the optimal estimate is the nonlinear weighted average of two terms: one estimate derived under the assumption that two inputs originate from a single source (fusion) and one derived under the assumption that they have separate sources (segregation), with each estimate weighted by the probability of the respective causal structure. This strategy is known as “model averaging” (Fig. 1C).

**Fig 2:**
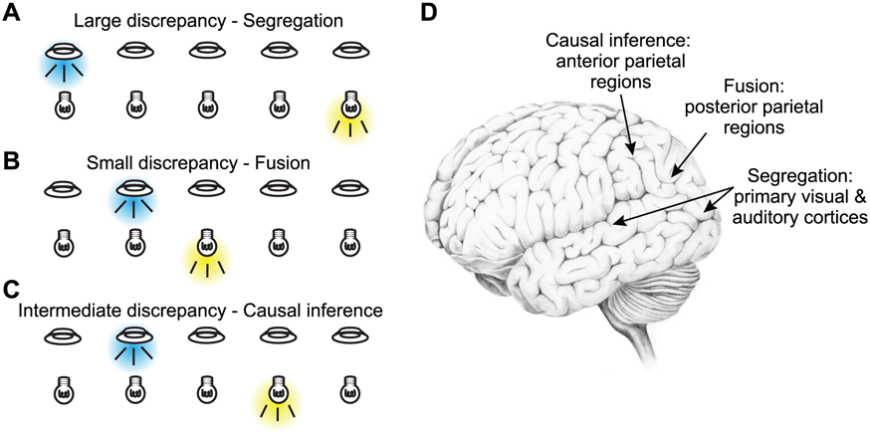
Causal inference about stimulus location. (A–C) Schematized spatial paradigm employed by several studies on audio-visual causal inference. Brief and simple visual (flashes) and auditory (noise bursts) stimuli are presented at varying locations along azimuth and varying degrees of discrepancy across trials. When stimuli are presented with large spatial discrepancy (panel A), they are typically perceived as independent events and are processed separately. When they are presented with no or little spatial discrepancy (panel B), they are typically perceived as originating from the same source and their spatial evidence is integrated (fused). Finally, when the spatial discrepancy is intermediate (panel C), causal inference can result in partial integration: the perceived locations of the two stimuli are pulled towards each other but do not converge. Please note that the probability distributions corresponding to each panel are shown in the respective panels in Fig. 1. (D) Schematized summary of the results by Rohe and Noppeney. Early sensory areas mostly reflect the unisensory evidence corresponding to segregated representations, posterior parietal regions reflect the fused spatial estimate, and more anterior parietal regions reflect the overall causal inference estimate. This distributed pattern of sensory representations demonstrates the progression of causal inference computations along the cortical hierarchy.

In testing whether Bayesian causal inference can account for human perception, localization tasks have been proved useful (Fig. 2) [6,17–19]. When visual and acoustic stimuli are presented with a small spatial disparity (Fig. 2A), they are likely perceived as originating from the same source, hence their evidence about the spatial location should be fused. In contrast, if their spatial disparity is large (Fig. 2B), the two inputs likely arise from distinct sources and should be segregated. Interestingly, the model also predicts a more surprising behaviour in conditions in which spatial disparity is moderate (Fig. 2C). In this case, the two senses get partially integrated, weighted by the relative likelihood of one or two origins [17]—assuming that the brain adopts a model averaging strategy, which seems to be the case in many observers [19]. Therefore, Bayesian causal inference successfully explains perceptual judgements across the range of discrepancies, spanning a continuum, from fusion to partial integration to segregation.

Again, its success in describing human perception suggests that this Bayesian model could also provide a framework to map the underlying neural processes onto distinct sensory computations. For example, an important question is whether the same or distinct brain regions reflect the integration process and the causal inference computation. This is precisely what the study by Rohe and Noppeney addressed.

## Mapping Causal Inference onto Sensory Pathways

In their study, participants localized audiovisual signals that varied in spatial discrepancy and visual reliability while their brain activity was measured using functional magnetic resonance imaging (fMRI). The authors first fit the causal inference model to the perceptual data, which enabled them to investigate the mapping between brain activity and the different spatial estimates predicted by the model; the estimates were predicted by either unisensory input (corresponding to the distinct causal origins hypothesis), by the fusion of the two sensations (corresponding to the single causal origin hypothesis), or by the causal inference model (the weighted combination of fusion and segregation). Addressing this question required an additional step of data analysis: linking the selectivity to spatial information reflected in distributed patterns of fMRI activity to the spatial estimates predicted by each model component. Luckily, methods of decoding analysis provide a means to establish such a link [20] and allowed the authors to associate each brain region of interest with the best-matching sensory estimate predicted by the inference model.

As may be expected, some regions (early visual and auditory cortices) predominantly reflected the unisensory inputs and hence were only a little affected by any multisensory computation (see Fig. 2D). Other regions, e.g., those involved in establishing spatial maps (posterior regions in the intraparietal sulcus), reflected the fused estimate. Thus, in these regions, automatic integration processes seem to occur that merge the spatial evidence provided by different modalities, weighted by their reliability, but regardless of how likely it is that these relate to the same object. And finally, regions more anterior in the intraparietal sulcus encoded the spatial estimate as predicted by the causal inference model, hence adapting their sensory representation based on the likely causal origin.

Overall, the new results show that different neural processes along the sensory pathways reflect distinct estimates about the localization of sensory events. Some estimates seem to arise mostly in a simple unisensory manner, while others exhibit the computationally complex nature required for causal inference. In addition, they suggest that sensory fusion and causal inference, at least in the context of spatial perception, are distributed processes not necessarily occurring in the same regions. And finally, they reveal a gradual emergence of multisensory computations along “visual” pathways. The data support both the traditional notion that multisensory perception is mostly implemented by higher-level association regions and the more recent notion that early sensory regions also participate in multisensory encoding [21,22]. Most importantly, however, they show how model-driven neuroimaging studies allow us to map complex sensory operations such as causal inference onto the sensory hierarchies.

One conclusion from this and previous studies is that multisensory perception does not result from a single and localized process—that would fit the often and sometimes abused term “multisensory integration” [23]. Rather, multisensory perception arises from the interplay of many processes and a network of interacting regions that implement these, each possibly relying on a different assumption about the causal structure of the environment and implementing a different sensory computation. Ultimately, it may be impossible to fully understand localized multisensory processes without considering them in the big picture of a possibly hierarchical but certainly distributed organization.

## Conclusions

As with any major step forward, the results pose many new questions. For example, judging the environment’s causal structure relies on prior knowledge and experience [7,12], but we don’t know whether the processes of causal inference and incorporating prior information are implemented by the same neural processes. It will also be important to see whether there are brain regions generically involved in multisensory inference and not specific to spatial attributes. Furthermore, it seems natural to look for similar gradual changes in multisensory computations along other sensory pathways. For example, our understanding of auditory pathways may benefit from such model-based decoding studies [24]. Finally, the roles of attention and task relevance for multisensory perception remain controversial. Attentional selection modulates multisensory integration, and multisensory coincidences attract attention and amplify perception [25]. It remains unclear how attentional state or task relevance influence which sensory variables are represented in any brain region, and recent studies reveal complex patterns of mixed selectivity to task- and sensory-related variables in higher association regions [26]. Disentangling the impact of attention and task nature on multisensory encoding and what can actually be measured using neuroimaging signals remains a challenge for the future.

Neuroimaging studies provide critical insights into the large-scale organization of perception, but the underlying local mechanisms of neural population coding remain to be confirmed. Signatures of multisensory encoding at the single neuron level can be subtle [27], and the mixed selectivity of higher-level sensory regions can render the link between neural populations and neuroimaging ambiguous [28]. Again model-driven approaches may help, for example, by providing testable hypothesis about large-scale population codes that can be extracted from electrophysiological recordings or neuroimaging [14].

On a methodological side, recent work has shown how combining fMRI with probabilistic models of cognition can be a very powerful tool for understanding brain function [29,30]. In line with this, Rohe and Noppeney show that the combination of statistical models of perception and brain decoding has the power to enlighten our understanding of perception far beyond more descriptive approaches. Yet, studies such as this require carefully crafted models and efficient paradigms to overcome the poor signal-to-noise ratio sometimes offered by neuroimaging. As a result, further advances in both perceptual models and signal understanding and analysis are required to eventually uncover why we sometimes benefit from seeing a speaker in a noisy environment and why we get fooled by the ventriloquist’s puppet.
